# Low-Temperature Bonding for Heterogeneous Integration of Silicon Chips with Nanocrystalline Diamond Films

**DOI:** 10.3390/mi15121436

**Published:** 2024-11-28

**Authors:** Jicun Lu, Xiaochun Lv, Chenghao Zhang, Chuting Zhang, Yang Liu

**Affiliations:** 1 Guanghua Lingang Engineering Application Technology Research and Development (Shanghai) Co., Ltd., Shanghai 201306, China; zhangct2023@163.com; 2 School of Materials Science and Chemical Engineering, Harbin University of Science and Technology, Harbin 150080, China; lvxiaochun@163.com (X.L.); yang_liu@hrbust.edu.cn (Y.L.)

**Keywords:** diamond/Si bonding, copper paste, low-temperature sintering, Ti/Cu depositing, interface structure

## Abstract

Integrating nanocrystalline diamond (NCD) films on silicon chips has great practical significance and many potential applications, including high-power electronic devices, microelectromechanical systems, optoelectronic devices, and biosensors. In this study, we provide a solution for ensuring heterogeneous interface integration between silicon (Si) chips and NCD films using low-temperature bonding technology. This paper details the design and implementation of a magnetron sputtering layer on an NCD surface, as well as the materials and process for the connection layer of the integrated interface. The obtained NCD/Ti/Cu composite layer shows uniform island-like Cu nanostructures with 100~200 nm diameters, which could promote bonding between NCD and Si chips. Ultimately, a heterogeneous interface preparation of Si/Ag/Cu/Ti/NCD was achieved, with the integration temperature not exceeding 250 °C. The TEM analysis shows the closely packed atomic interface of the Cu NPs and deposited Ti/Cu layers, revealing the bonding mechanism.

## 1. Introduction

Throughout the development of modern electronic industry, the density and power of electronic devices has kept increasing, raising the requirements for electronic packaging materials and structures, such as their thermal management, mechanical support, and stability requirements [1,2,3]. Electronic packaging substrates, made from ceramics, organic laminates, or metals, are critical components in the electronics industry, providing mechanical support and electrical interconnections for integrated circuits [4,5]. Of the different packaging substrates available, ceramic substrates can withstand thermal cycling, vibration, and other environmental stresses while maintaining signal integrity and heat dissipation [6,7]. For example, Al_2_O_3_, AlN, and Si_3_N_4_ ceramics have been widely applied in electronic packaging due to their excellent thermal conductivity, mechanical strength, and dielectric properties [8,9,10]. Compared with ceramics, diamonds have higher thermal conductivity, higher insulation, excellent chemical stability, and superior mechanical properties, implying that they have good prospects in the field of electronic packaging [11,12].

Integrating diamond substrates and Si chips can lead to excellent heat dissipation performance, and a reliable bond between diamond and Si chips is required for practical application [13]. Bonding diamond to other materials always relies on the use of a metal modification layer, such as Ni, Cu, or Ag [14,15]. However, diamonds and metals have significantly different crystalline structures and properties, making it challenging to join them directly. Thus, an active metal layer, such as Cr and Ti, is always prepared on the diamond to improve the adhesion force, followed by Cu or Ag deposition [16,17]. To realize the bonding of diamonds to semiconductors, Zhong [18] described the low-temperature connection of diamonds to Si chips using Ti and Cu intermediate nanolayers. The bonding layer exhibited good mechanical properties and thermal conduction.

Recent advancements in low-temperature bonding technology have revolutionized semiconductor packaging. The realization of bonding at a temperature below 300 °C relies on the surface treatments used and the development of novel interlayer materials. By integrating diverse materials and components, low-temperature bonding technology could drive future innovations in electronics and photonics, and the contact surfaces for bonding could be metal or nonmetal [13,14,19]. The Cu pillars prepared on the surface could achieve low-temperature bonding, and a passivation layer or surface activation could further promote bond formation, including Pd, Pt, and Au layers [20,21]. In actual applications, using Cu or Ag sintering, depending on the nanoparticle (NP) used, is a good choice for packaging and bonding [22,23,24,25]. The high surface energy levels of NPs could lead to low-temperature bonding, forming a stable connection [26]. For instance, pressure-assisted Ag sintering has been demonstrated for the bonding of diamond and Si, with a thin Cr/Au layer deposited on the surface in advance [27]. Compared with Ag sintering, Cu NP sintering has better application prospects due to its low cost and lack of ion migration [28]. In previous studies, Cu NPs formed a high-strength bond at a temperature lower than 300 °C [25,29]. To further improve the properties of sinter joints, Cu particles with multi-level hierarchical structures have been proposed for sintering [30]. The solvent composition of a Cu paste has also been altered to optimize the sintering process [31,32]. These efforts have broad prospects for Cu sintering in the field of packaging. The Cu-sintered layer has outstanding thermal conductivity, electrical conductivity, and mechanical strength properties [33,34]. However, up to now, limited research has been conducted on the bonding of diamond and Si chips based on Cu NP sintering.

In this study, magnetron sputtering was first used to deposit Ti and Cu layers on a nanocrystalline diamond (NCD) film. The morphology and phase composition of the Ti/Cu layer were carefully characterized and discussed. After Ti/Cu deposition, the NCD layer was bonded with a Si chip via a thermo-compression Cu NP sintering step. The sintering temperatures’ effect on the interfacial microstructures was investigated. The interfacial microstructures and sintering mechanism of the joint were also comprehensively analyzed.

## 2. Experimental

### 2.1. Materials

Si chips with a metallization layer of Ti/Ni/Ag were purchased from Innotronix Technology Company (Beijing, China). Cu NPs (~100 nm and ~200 nm) prepared via in situ self-reduction were purchased from Guangzhou Hongwu Material Technology Company (Guangzhou, China). Ethanol (C_2_H_6_O, 99.7%), glycerol (C_3_H_8_O_3_, 99.5%), and L-ascorbic acid (C_6_H_8_O_6_, 99%) were purchased from Sigma Aldrich (Shanghai, China). All reagents were used without further purification.

### 2.2. Magnetron Sputtering and Thermo-Compression Sintering

**Figure 1:**
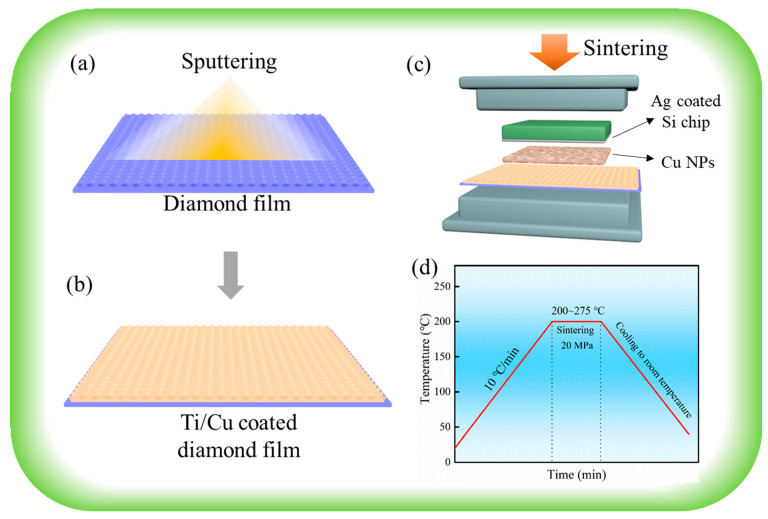
Schematic diagram of (**a**) sputtering on diamond film, (**b**) Ti/Cu-coated diamond film, (**c**) sintering, and (**d**) heating curve.

The NCD film was deposited on polished Si via hot-filament chemical vapor deposition (CVD). The reactive gasses used in the CVD process were CH_4_ and H_2_ with flow rates of 6 and 300 SCCM, respectively. An NCD sample with a thickness of ~200 nm was treated via Ti deposition, followed by Cu deposition via magnetron sputtering (shown in Figure 1a), achieving an NCD/Ti/Cu structure (shown in Figure 1b). The sputtering power was 60 W. The deposition times for Ti and Cu were 30 min and 90 min, respectively.

The as-prepared Ti/Cu-coated NCD sample was used as the substrate for the thermo-compression sintering, as shown in Figure 1c. A Cu paste was formed by mixing Cu NPs (~100 nm and ~200 nm), glycerol, and L-ascorbic acid, which were ground in the three-roller machine for 3 h. The weight ratio of glycerol and L-ascorbic acid was 20:1. The Cu paste with a thickness of 120 μm was prepared on the NCD/Ti/Cu sample by using screen printing. Then, the Si chip was placed on the as-printed Cu paste, obtaining the sintering assembly. Figure 1d shows that sintering was conducted at 20 MPa for 10 min. The heating temperature ranged from 200 °C to 275 °C, with a heating rate of 10 °C/min.

### 2.3. Morphology Characterization and Strength Test

The morphology of the Ti/Cu layer on NCD and the cross-sectional interface of sintered joints were observed using scanning electron microscopy (SEM, Merlin Compact, Zeiss, Oberkochen, Germany). The phase compositions of the NCD sample with a Ti/Cu layer were analyzed via X-ray diffraction (XRD, D8 ADVANCE, Bruker, Karlsruhe, Germany). The interfacial bond was analyzed via transmission electron microscopy (TEM, Talos f200×, FEI, Waltham, MA, USA), and the TEM sample was prepared using FIB (ThermoFisher, V400ACE, Waltham, MA, USA). An atomic force microscope (AFM, Dimension Fastscan, Bruker, Karlsruhe, Germany) was used to measure the surface roughness.

## 3. Results and Discussion

### 3.1. Ti/Cu Sputtering on the NCD Surface

**Figure 2:**
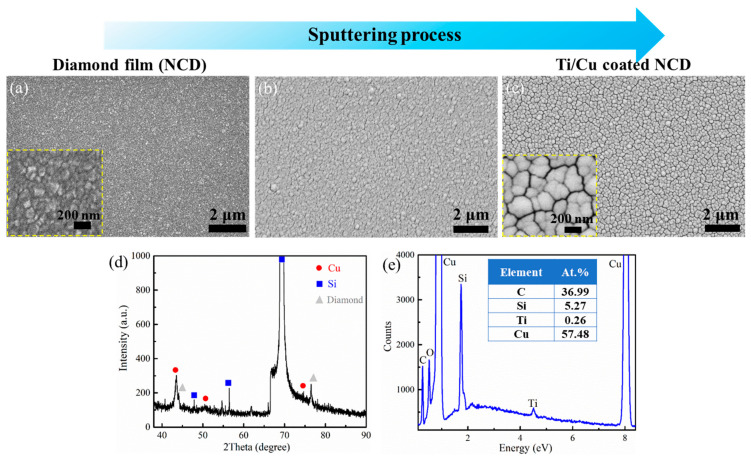
SEM images of NCD films (**a**) before sputtering, (**b**) during the sputtering process and (**c**) after sputtering Ti/Cu, (**d**) XRD and (**e**) EDS results of the Ti/Cu-coated NCD.

The morphology of the prepared NCD film is shown in Figure 2a. Diamond grains are distributed uniformly on the surface, and the size of most grains is smaller than 100 nm. The NCD sample was treated using piranha solution, which promoted the adhesion force for the metal deposition that followed. In principle, piranha solution can remove organic groups and introduce an -OH group to the sample surface [35,36]. The SEM micrographs of the surface morphology during and after the sputtering process are shown in Figure 2b,c, respectively. Figure 2b demonstrates that the morphology is similar to that of the as-prepared diamond grains shown in Figure 2a. However, the size increases and the surface becomes smooth, unlike in Figure 2a.

The sample shown in Figure 2c has high-density island-like nanostructures of approximately 100 nm to 200 nm. An island-like structure can be regarded as the aggregation of several nanoparticles. Gaps surrounding the island-like nanostructures can be distinguished. The appearance of island-like structures on the surfaces of magnetron-sputtered films can be explained as follows: In the early stages of film deposition, individual atoms arriving on the substrate aggregate into small clusters, or “islands”, to minimize the surface energy. As more material is deposited, the Volmer–Weber growth mode enables island formation [37]. The rough surfaces of NCD films, resulting from the diamond nano-grains, also contribute to the formation of island-like structures. When the island-like structures reach a certain height, the top of the islands obstruct the individual atoms from arriving at the lower parts of the substrate, forming the “gap” parts. The deposition rate on the island is larger than that within the gaps, further accelerating the growth of island-like structures on the sample surface [38]. Using AFM measurements, the low arithmetic mean surface roughnesses (Ras) of the as-deposited, piranha-treated, and Ti/Cu-sputtered NCD surfaces are 6.4 nm, 7.0 nm, and 9.2 nm, respectively. The piranha treatment does not notably influence the surface roughness due to the chemical stability of the diamond. The surface roughness increases after Ti/Cu deposition.

The XRD patterns of the NCD sample after sputtering are shown in Figure 2d. The peaks of Si, diamond, and Cu reveal the successful deposition of the Cu layer on diamond film. Figure 2e shows the EDS spectra of the NCD sample after sputtering. The proportions of C, Si, Ti, and Cu are 36.99%, 5.27%, 0.26%, and 57.48%, respectively. C can be attributed to the NCD and the carbon absorbed from the air. O originates from absorbed oxygen-related groups and the minor oxidation of the Cu deposition layer. O is not taken into account when calculating atomic percentages. The content of Ti is low because its deposition time is relatively short, and the Ti layer is below the Cu layer. The absence of Ti in the XRD-related result is attributed to the low thickness of the Ti layer (shown in Figure 2d).

**Figure 3:**
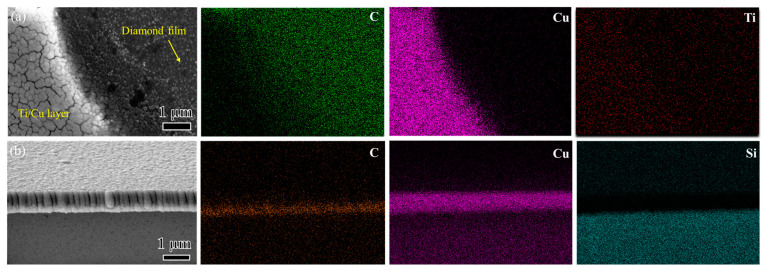
(**a**) Top-view SEM image and element maps of the boundary region of Ti/Cu coating on NCD, (**b**) cross-section view SEM and element maps of the Ti/Cu coating on NCD.

Part of the NCD surface is protected by a small piece of Si, acting as the hard mask during the sputtering process. Figure 3a shows a top-view SEM image and the corresponding element maps of the boundary region of the Ti/Cu layer on the NCD surface. Cu is mainly distributed in the left region of white island-like nanostructures, and the Ti content in the left region is also slightly larger than that in the right region, aligning with the Ti/Cu deposition layers. The right dark region corresponds to C, aligning with the NCD film without Ti/Cu deposition. A narrow region could be observed between the white and dark regions. Figure 3b displays the cross-sectional SEM image and corresponding element maps. The deposited Cu layer with a thickness of 0.8 μm can be observed on the NCD film. Si corresponds to the Si substrate of the NCD. The Ti layer is not apparent due to its small thickness.

Therefore, the analysis above proves that the Ti/Cu bilayer is successfully fabricated on the NCD film, with Cu island-like nanostructures present on the surface. Moreover, the island-like nanostructures can maintain a high surface area and surface energy, which is profitable for the sintering process outlined below.

### 3.2. Interfacial Microstructure and Mechanism of the Interfacial Bonding

**Figure 4:**
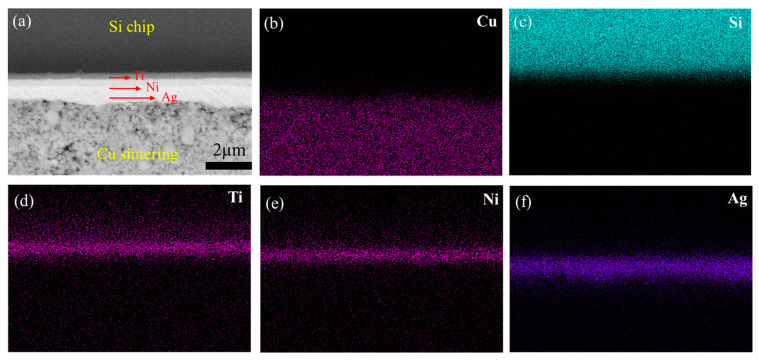
(**a**) Interfacial microstructures of the Si chip and sintered Cu NPs, the corresponding element map distribution of (**b**) Cu, (**c**) Si, (**d**) Ti, (**e**) Ni, and (**f**) Ag.

After Ti/Cu deposition, the as-obtained NCD/Ti/Cu sample is sinter-bonded with a Si chip. The sample sintered at 250 °C is then analyzed. Figure 4a shows the SEM image of the Si chip/sintered Cu interface. The corresponding element map distribution is displayed in Figure 4b–f. Three thin layers, Ti, Ni, and Ag, are arranged from top to bottom in sequence. The Ag layer is directly bonded to the sintered Cu. This indicates that the Si chip/Ti/Ni/Ag/Cu NPs structure is achieved at the side of the Si chip.

**Figure 5:**
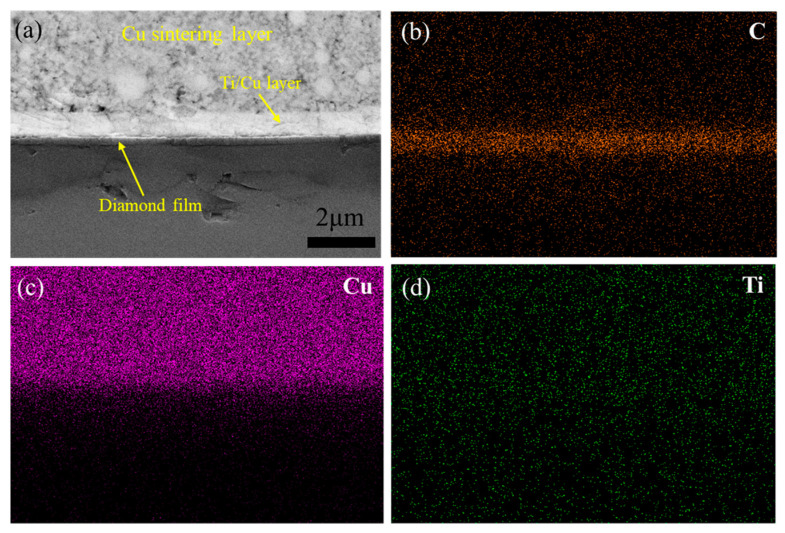
(**a**) Interfacial microstructures of the NCD film and sintered Cu NPs, the corresponding element map distribution of (**b**) C, (**c**) Cu, and (**d**) Ti.

Figure 5a presents an SEM image of the sintered Cu/diamond interface, and the corresponding element map distribution is shown in Figure 5b,d. Cu can be attributed to the sintered Cu NPs and the Cu deposition layer. Ti is not clearly shown due to its low thickness (Ti will be analyzed using TEM images). Thus, the NCD/Ti/Cu/Cu NPs sintered structures can be seen at the side of the NCD interface.

**Figure 6:**
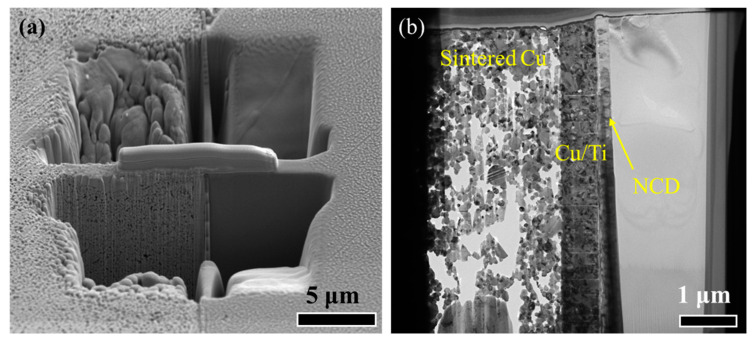
(**a**) The FIB processing for preparing the TEM sample, (**b**) as-prepared sample for TEM observation.

To further investigate the interfacial bonding between NCD/Ti/Cu and the Si chip, the sample is cut by FIB, as shown in Figure 6a. After the ion-beam thinning treatment, the as-prepared TEM sample, as displayed in Figure 6b, shows the sintered Cu NPs, Cu/Ti bilayer, and NCD film. We find that the pores in Figure 6b are much larger than those in Figure 6a due to effect of the FIB.

**Figure 7:**
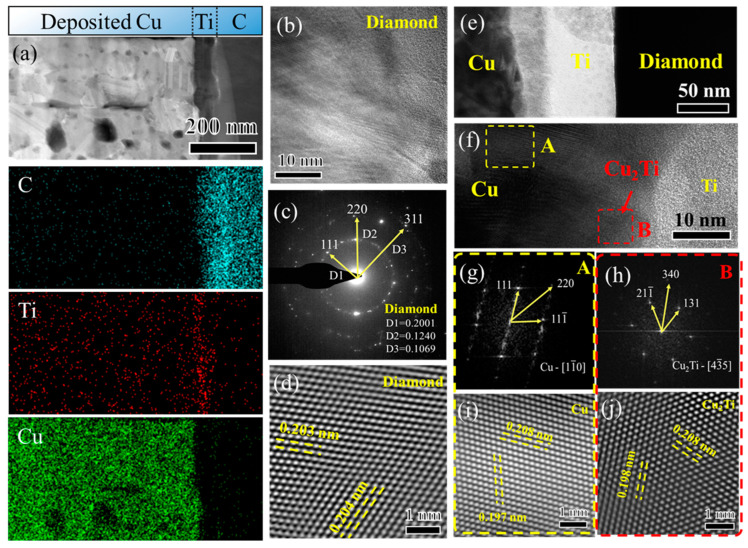
TEM analysis of the NCD/Ti/Cu interface. (**a**) STEM image and the corresponding element map distributions, (**b**) HRTEM images of the NCD, (**c**) FFT patterns of the NCD, (**d**) IFFT image of the NCD, (**e**) bright-field image of Cu/Ti/NCD film, (**f**) HRTEM images of the interface deposited Cu/Ti layer, FFT patterns of (**g**) Region A and (**h**) Region B marked in (**f**), IFFT images of (**i**) Region A and (**j**) Region B.

Figure 7a presents the STEM image and the corresponding element maps of the deposited Ti/Cu layer on NCD. The Cu, Ti, and C distributions indicate the interface of deposited Cu/Ti/diamond. Figure 7b displays a high-resolution transmission electron microscopy (HRTEM) image of the diamond film. The FFT (Fast Fourier Transform) patterns taken from Figure 7b are shown in Figure 7c, aligning with those of polycrystalline diamond. Figure 7d shows the IFFT (Inverse Fast Fourier Transform) image of a diamond. Figure 7e demonstrates the interface of Cu/Ti/NCD, confirming that the Ti layer is tightly bonded with NCD and the deposited Cu layer. Ti serves as an intermediate layer to ensure a stable connection between NCD and the deposited Cu layer. The HRTEM image of the Cu/Ti bilayer interface is shown in Figure 7f. The FFT patterns and IFFT image of Region A (shown in Figure 7f) are displayed in Figure 7g,i, respectively. The (111) plane of Cu can be identified, and the FFT patterns can be identified as Cu using the zone axis of [1$\overset{-}{1}$0]. For Region B marked in Figure 7f, the FFT pattern (Figure 7h) and HRTEM image (Figure 7j) point to the presence of Cu_2_Ti at the interface of the deposited Cu/Ti bilayer.

**Figure 8:**
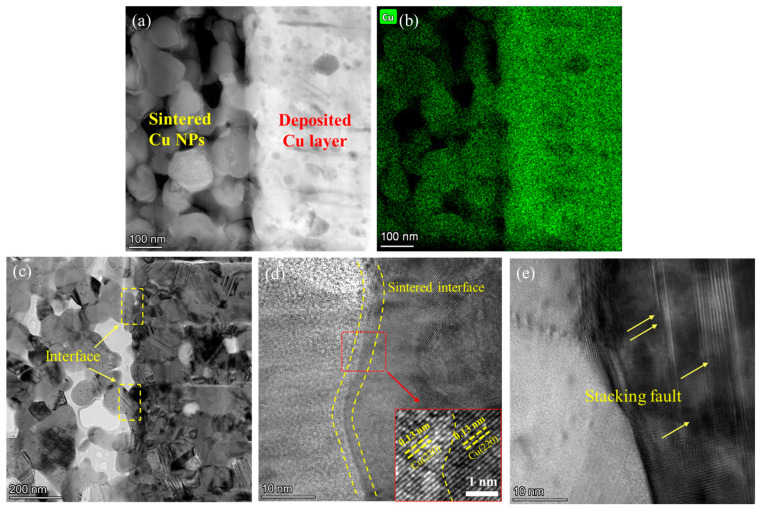
TEM analysis of the interface between deposited Cu layer and sintered Cu NPs. (**a**) STEM image, (**b**) corresponding Cu element map distribution, (**c**) bright-field image, (**d**) HRTEM image of the interface, (**e**) the stacking faults in the deposited Cu layer.

The STEM image of the interface of the sintered Cu NPs and deposited Cu layer is shown in Figure 8a. Cu NPs are well bonded to the deposited layer, forming a non-continuous bonding interface. Only Cu can be clearly detected at the interface, as shown in Figure 8b. Figure 8c is the bright-field image of the interface of the sintered Cu NPs and deposited Cu layer. The bonding interface is similar to that in Figure 8a. Notably, the deposited Cu layer is composed of numerous grains with sizes smaller than 200 nm, contributing to the bonding to Cu NPs. Figure 8d presents the HRTEM image of the Cu NP/deposited Cu interface, and the inset image shows the high magnification of the selected region. The Cu (220) planes can be identified at both the sintered Cu NPs and deposited Cu layer, illustrating reliable atomic bonding. Interestingly, the stacking faults shown in Figure 8e offer a high energy state for the deposited Cu, promoting sintering [39].

**Figure 9:**
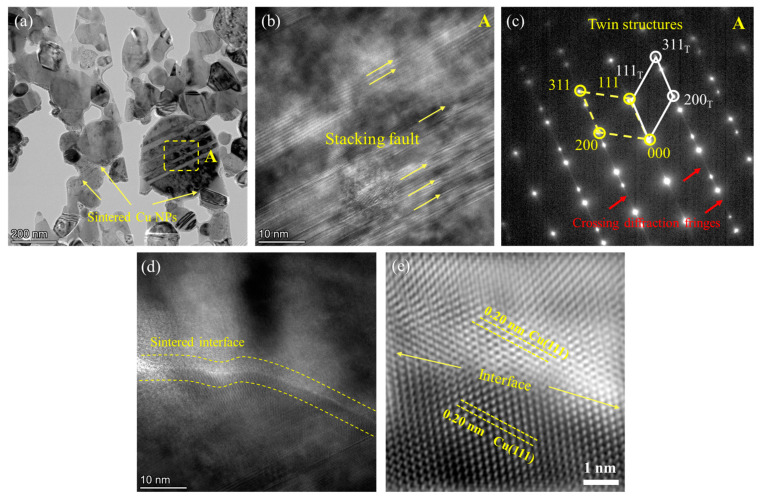
TEM analysis of the interface between sintered Cu NPs. (**a**) Bright-field image, (**b**) HRTEM image, and (**c**) SAED patterns corresponding to Region A in (**a**), (**d**) HRTEM image of the interface, (**e**) magnified image of (**d**).

The morphologies of the sintered Cu NPs are shown in Figure 9a, in which nanoparticles with sintered necks can be observed. Figure 9b is the HRTEM image of Region A located within a particle shown in Figure 9a, where many stacking faults can be observed. Figure 9c is the SAED pattern corresponding to Region A of a Cu NP. Crossing diffraction fringes between the spots confirm the stacking faults in the nanoparticle. On the other hand, twin structures can be found; this localized distortion in the stacking faults increases the system’s internal energy. Twin crystals can reduce the system’s energy by providing low-energy interfaces that facilitate atomic diffusion. In principle, stacking faults and twin boundaries can serve as active sites during the sintering process, facilitating mass transport. In the early stages of sintering, these defective regions possess higher energy levels, which lower the activation energy and provide rapid channels for atomic diffusion, accelerating atom migration and rearrangement, thereby promoting sintering [39]. The presence of these factors can modify the local stress fields and energy landscape, promoting grain growth and improving the sintered joint’s mechanical properties. From Figure 9d, the Cu NP interface after sintering can be observed. The magnified image of the interface region is displayed in Figure 9e. The Cu (111) planes can be identified on both sides of the interface. This confirms that two Cu NPs are closely packed at the atomic scale, ensuring the reliable sintered bonding of the Cu NPs.

### 3.3. Effect of the Sintering Temperature on the Interfacial Bonding

**Figure 10:**
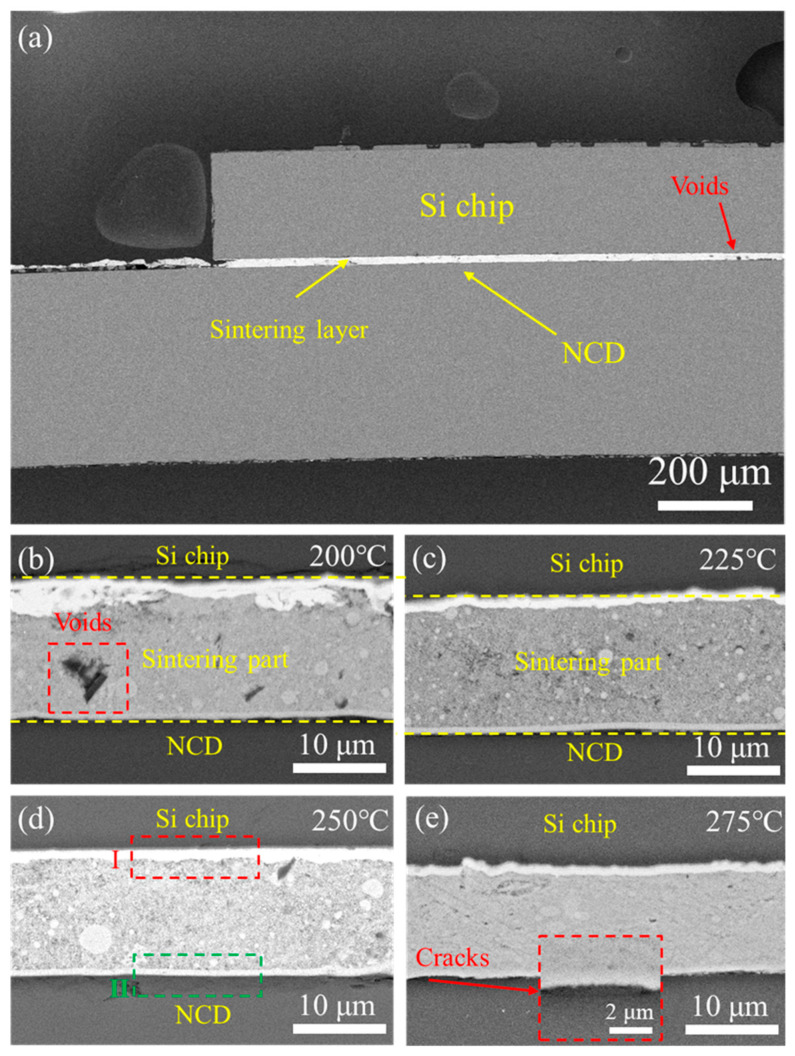
(**a**) Low-magnification SEM image of the sintered joint at 200 °C. Cross-sectional images of the joints sintered at (**b**) 200 °C, (**c**) 225 °C, (**d**) 250 °C, and (**e**) 275 °C.

The sintering temperature plays an important role in bonding. The acceptable temperature for Si chip bonding is below 300 °C, and a temperature over 200 °C is always required for achieving stable sintered joints. Thus, sintering temperatures of 200 °C, 225 °C, 250 °C, and 275 °C are set to study their effects on the bonding interface. Figure 10a illustrates the low-magnification cross-sectional image of the joint sintered at 200 °C. We observe that a uniform seam is formed between the Si chip and the NCD sample via the thermo-compression sintering of the Cu NPs. Some small voids can be found at the sintered Cu layer. Figure 10b–e display the interfacial microstructures of the joints sintered at 200 °C, 225 °C, 250 °C, and 275 °C, respectively.

Overall, the Si chip is well bonded with the NCD sample, constructing the Si chip/sintered Cu NPs/Cu/Ti/NCD joints. When the sintering temperature is 200 °C, the evident voids in the joint can be attributed to inadequate sintering and the effect of glycerol, as shown in Figure 10b. The evaporation of glycerol and its decomposition products can introduce voids in the joint during sintering. As the sintering temperature increases, the number of voids decreases, as shown in Figure 10c,d. When the sintering temperature is 250 °C, good bonds can be observed at the Cu NP/Si chip and Cu NP/diamond interfaces, as shown in Regions I and Ⅱ, respectively. Increasing the temperature can promote sintering, enhance the density of the sintered structure, and reduce defects. When the sintering temperature reaches 275 °C, the thickness of the sintering layer decreases and the sintered microstructure becomes denser. However, a few interface cracks appear at the interface between the Ti/Cu layer and the NCD film, as shown in Figure 10e, most likely due to coefficient of thermal expansion (CTE) mismatch. The CTE values of Cu, Si and, diamond are 1.7 × 10^−5^/°C [40], 2.33 × 10^−6^/°C [41], and 0.8 × 10^−6^/°C [42], respectively. A higher heating temperature could lead to higher CTE mismatch and higher residual stress during the cooling step.

The obtained results demonstrate the feasibility of the heterogeneous integration of Si chips on diamond substrates. Ti/Cu deposition and low-temperature bonding of Cu NPs provide a new method for using diamond substrates in electronic packaging. The packaging structures may have many positive properties in real-world applications, such as high bonding strengths, good thermal management, and good insulation. Additionally, bonding at a temperature below 200 °C could be studied in a follow-up study to further expand the application region.

## 4. Conclusions

In summary, we developed a novel method for integrating nanocrystalline diamond (NCD) with Si chips. The joints of the Si chip and diamond were bonded through Ti/Cu deposition and Cu NP sintering. The Ti/Cu bilayer was successfully fabricated on the NCD surface via magnetron sputtering at 60 W, forming the NCD/Ti/Cu structure. Cu nanostructures with sizes of ~200 nm formed an island-like shape and were distributed uniformly on the sample surface, contributing to thermo-compression sintering. A uniform and reliable seam was obtained between the Si chip and NCD based on the in situ self-reduction sintering of multi-sized nano Cu. From the TEM analysis, we concluded that the Cu NPs formed a tight bond with the Ti/Cu deposition layer at a temperature not exceeding 250 °C. Heterogeneous interface integration was achieved between silicon (Si) chips and NCD films using low-temperature bonding technology.

This study realized the heterogeneous integration of Si chips and diamond substrates, providing a new option in the field of high thermal conductivity and insulation. Further studies may explore the properties of the sintered joints between Si chips and diamond substrates, such as the thermal conductivity and bonding strength.

## Data Availability

The raw data supporting the conclusions of this article will be made available by the authors on request.
